# Role of surface terminations on MXenes’ stability and performance in tribological applications

**DOI:** 10.1557/s43577-026-01157-0

**Published:** 2026-09-03

**Authors:** Enrico Pedretti, Andreas Rosenkranz, Maria Clelia Righi

**Affiliations:** 1 Department of Physics and Astronomy “Augusto Righi”, University of Bologna, Bologna, Italy; 2 Department of Chemical Engineering, Biotechnology and Materials, FCFM, University of Chile, Santiago, Chile; 3 ANID – Millennium Science Initiative Program, Millennium Nuclei of Advanced MXenes for Sustainable Applications (AMXSA), Santiago, Chile

**Keywords:** 2D materials, Chemical synthesis, Coating, Layered, Surface chemistry, Tribology

## Abstract

MXenes, a growing class of two-dimensional transition-metal carbides, nitrides, and carbonitrides, have become increasingly relevant in many fields, ranging from energy storage to biology and tribology, due to their excellent mechanical and surface properties. MXenes are synthesized by selective etching processes, resulting in surface terminations, usually –O, –OH, and –F, whose relative abundance depends on the etching procedure adopted. In this article, we examine the effect of MXenes’ surface terminations on their stability and tribological performance, with a particular emphasis on recent advances in the synthesis routes, allowing for better control over the terminations’ composition and uniformity. We then discuss the stability of MXenes under different conditions, such as in solution or within coatings, summarizing experimental evidence for oxidation and hydrolysis in air and aqueous media, focusing on how surface and edge passivation influences degradation pathways and long-term stability. Subsequently, we focus on tribological applications, exploring MXenes behavior under tribological conditions, for which contact pressure, shear, and local temperature can activate tribochemical reactions, promote termination redistribution, and drive the formation of protective tribofilms that can reduce wear of the substrate. Finally, we analyze how surface terminations affect lubricity by modifying interlayer interactions, adhesion, and shear strength. By reviewing the most relevant results and clearly addressing the existing knowledge gaps, this article aims at summarizing the key design principles and outlining the remaining challenges to develop MXene-based solid lubricants and lubricant additives.

**Graphical abstract:**

**Figure:**
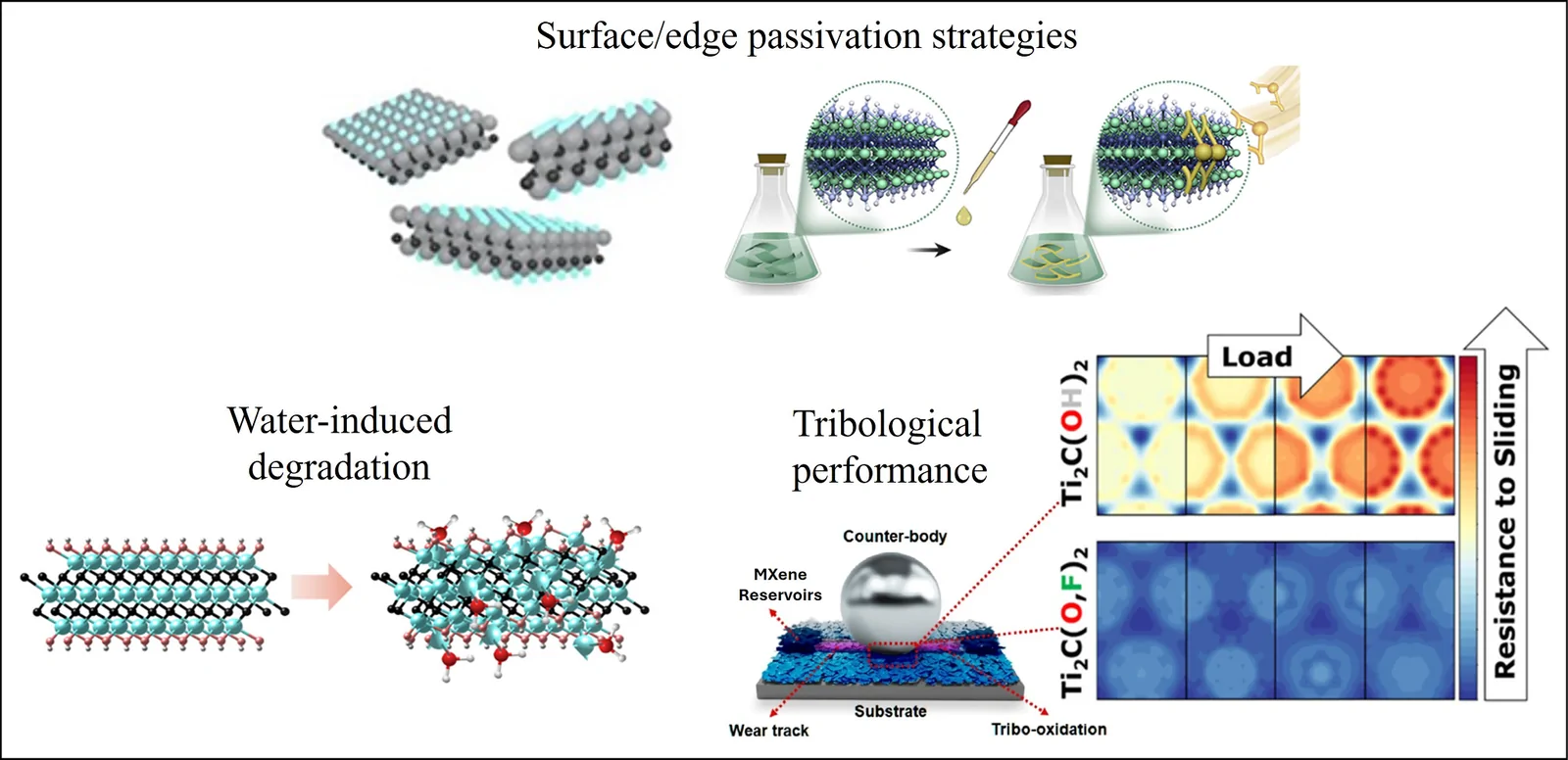

## MXenes synthesis and the effect of posttreatment methods on surface terminations

MXenes are a large and rapidly expanding family of two-dimensional (2D) transition-metal carbides, nitrides, and carbonitrides with the general formula M_n+1_X_n_T_x_ (n = 1–4), for which M represents an early transition metal (Ti, Nb, Mo, V, W, etc.), X denotes carbon and/or nitrogen, and T_x_stands for the surface terminations (such as –F, –O, –H, –OH, –Cl, –Br).^1^ This broad class of materials finds increasing interest in many fields, including energy storage,^2–4^ catalysis,^5,6^ electromagnetic shielding,^7^ flexible electronics,^8,9^ sensors,^10,11^ and tribology.^12^

MXenes are usually synthetized by selective etching of the A layer from three-dimensional (3D) crystalline MAX phase precursors (where A is usually a group 13/14 element, such as Al, Ga, Si).^13^ Upon etching, the freshly exposed and highly reactive transition-metal surfaces are immediately passivated by species from the surrounding environment, giving rise to surface terminations. These terminations constitute a fundamental component of the material, and their chemical identity, stoichiometry, and spatial distribution deeply affect MXenes’ physical and chemical properties, including their electrical conductivity, surface charge, resistance to degradation, as well as other optical, magnetic, mechanical, and tribological properties.^14^

**Figure 1:**
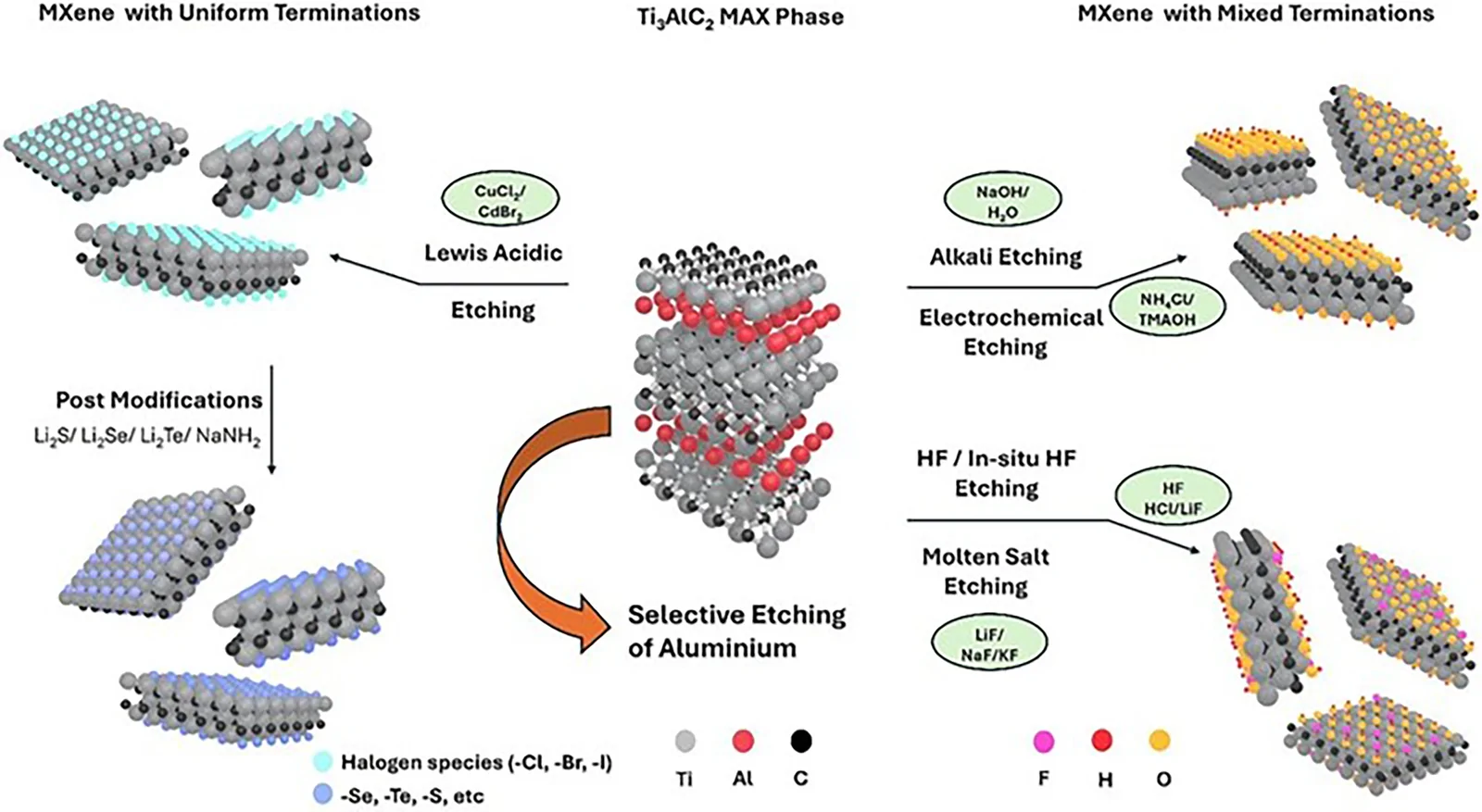
Main routes of MXenes synthesis starting from the MAX phase precursor. Selective etching of the A layers can be performed by multiple approaches, leading to different surface terminations. Hydrofluoric acid (HF) etching produces mixed –F, –O, –OH terminations, with relative abundance dictated by the HF concentration. Lewis acid molten salt etching enables a greater control and uniformity over surface terminations, resulting in halogen species (usually –Cl or –Br), which can then be substituted with other elements through postmodification methods. Other methods, such as alkali etching and electrochemical etching offer alternative strategies to avoid –F terminations, resulting in a combination of –O and –OH. Reprinted with permission from Reference 1. © 2025 Wiley.

As outlined in **Figure** **1**, several different etching approaches to obtain MXenes from MAX phases have been developed in the past decade, defined by the etchant type and concentration, environmental conditions, temperature and duration of the process, each affecting the resulting surface terminations.^15^ Moreover, posttreatment methods can be employed to further alter the composition of surface terminations after synthesis, allowing for improved control on MXenes’ properties. In the following paragraphs, we will briefly outline the main etching and posttreatment methods that have been reported up to date.

### Hydrofluoric acid-based etching: Mixed terminations

The original and most widely adopted route for MXene synthesis involves the selective etching of the A layer from the MAX precursors by dissolution in aqueous hydrofluoric acid (HF). The reaction yields mixture of –F, –OH, and –O terminations,^16^ whose relative abundance is dictated by the HF concentration, as the resulting higher H^+^concentration promotes the recombination of –O and –OH groups to water, leaving more surface vacancies to be filled by –F. For reference, Alhabeb et al. observed an increase of the fraction of –F terminations from 17 to 28% by varying HF concentration from 5 to 30%, respectively.^17^

Prolonged etching or excessively concentrated HF (>30%) does not merely shift the termination balance but also introduces structural defects (vacancies, broken bonds) that further alter the local coordination and stability of the terminations.^1^ A milder variant, *in situ* HF generation (e.g., LiF + HCl), produces a comparable mixed termination chemistry but yields a higher proportion of –O relative to –F and –OH, partly because the lower fluoride activity and subsequent vacuum drying favor the conversion of adjacent –OH pairs into –O and H_2_O^.1^ Moreover, the use of HCl can also introduce –Cl terminations.^18^ Despite its simplicity and scalability, the HF route inherently produces mixed and spatially non-uniform terminations, as confirmed by secondary ion mass spectrometry (SIMS) depth profiling, which shows –O and –OH enrichment on one face and –F enrichment on the opposite face of individual Ti_3_C_2_T_x_ sheets.^1^ This lack of compositional control is a fundamental limitation for applications that demand a well-defined surface chemistry.

### Lewis acid molten salt etching: Toward uniform terminations

**Figure 2:**
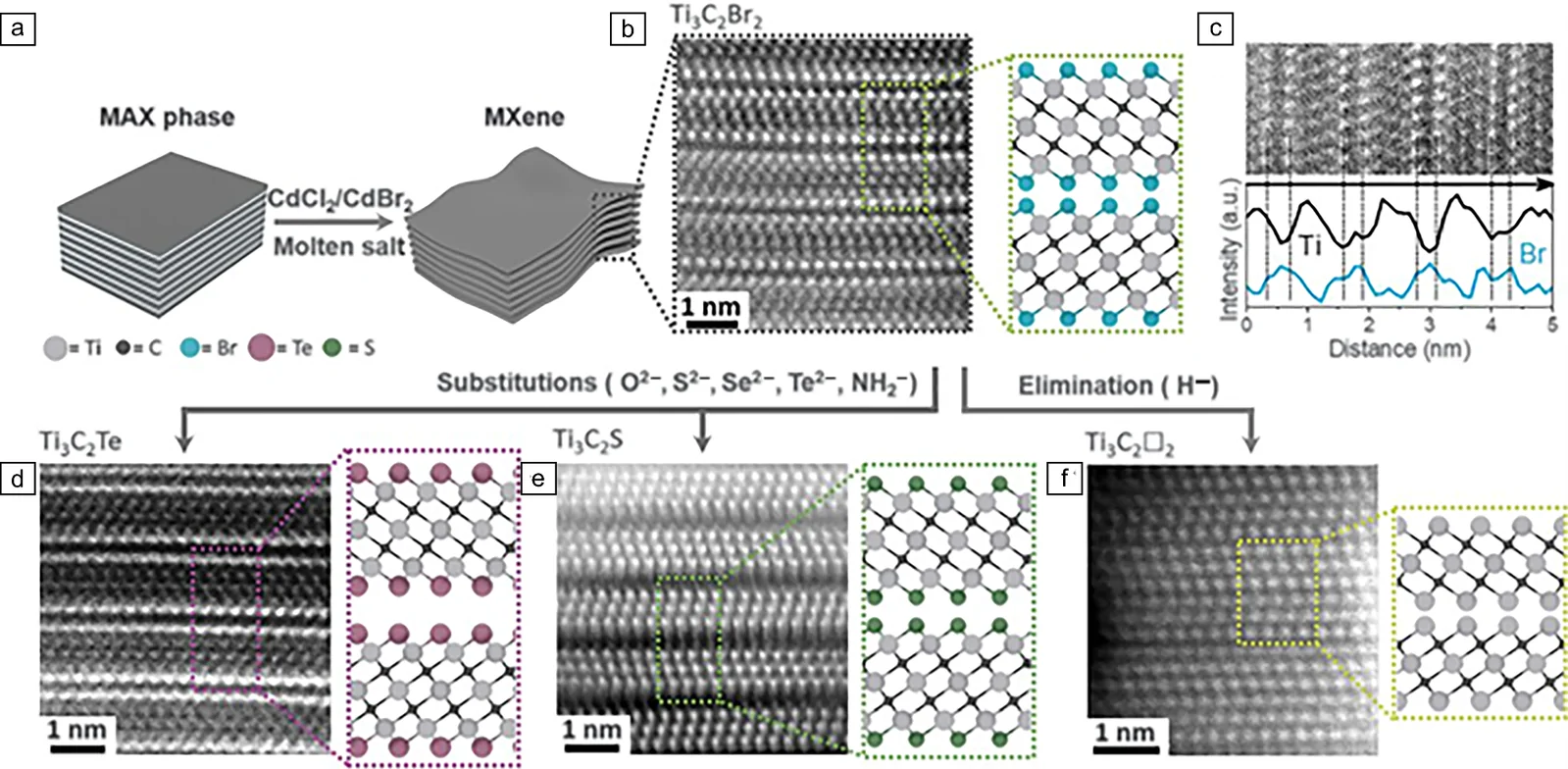
Lewis acid molten salt (LAMS) etching and post*-*synthesis modification to produce tunable surface terminations. (a) LAMS etching leads to –Br terminations (b) as shown by atomic*-*resolution high*-*angle annular dark*-*field (HAADF) image and (c) energy*-*dispersive x*-*ray elemental analysis, respectively. After synthesis, –Br can be substituted with (d) Te, (e) S and other elements, or (f) removed by reductive elimination, leaving T_x_ vacancies, as shown by the corresponding HAADF images. Reprinted with permission from Reference 21. © 2020 AAAS.

A transformative advance in termination control came with the introduction of Lewis acid molten salt (LAMS) etching, which allows for an improved control and tunability over terminations.^19^ In this approach, the A-layer of the MAX phase is replaced by a transition-metal cation from the molten salt (e.g., Zn^2+^ from ZnCl_2_), producing an intermediate cation-based MAX phase that subsequently reacts further to yield a MXene exclusively terminated by –Cl, such as Ti_3_C_2_Cl_2_ and Ti_2_CCl_2_.^20^ Performing this reaction above 500°C ensures that sufficient thermal energy is available for the anions to diffuse and occupy all available surface sites, yielding single-species –Cl terminations at the cost of any fluoride-related species.^20^ Extending the Lewis acid approach beyond chloride salts, Kamysbayev et al. demonstrated that Br-terminated MXenes (e.g., Ti_3_C_2_Br_2_) can be synthesized using CdBr_2_, and that the relatively weak M–Cl and M–Br bonds, weaker than M–F or M–O bonds, render these halogen-terminated phases highly versatile for further covalent surface chemistry.^21^ Through substitution reactions in molten inorganic salts that allow to replace and substitute surface groups, MXenes with –O, –NH, –S, –Se, –Br, –Te, and –Cl terminations, as well as bare (termination-free) MXene multilayers, have all been successfully prepared,^21,22^ as visible in **Figure** **2**. The molten salt strategy has thus notably expanded the accessible termination space beyond what any wet chemical route can achieve.

### Electrochemical etching, alkali etching, and alternative synthesis routes

Several additional synthesis strategies offer complementary termination profiles. The electrochemical etching of Ti_3_AlC_2_ in low-concentration HCl solution can produce MXenes with –Cl, –OH, and –O terminations, while the relative balance becomes tunable through the applied potential window and the choice of intercalant.^1^ Alkali etching employing an organic base (NaOH, KOH, TMAOH at 50–270°C) selectively yields –O and –OH terminations while avoiding fluoride entirely, though at the cost of lower universality and yield.^1^ Chemical vapor deposition (CVD) offers the unique ability to grow MXene films that are essentially bare, with no surface terminations, and allows for a precise thickness control, but currently suffers from limited scalability^1^ An alternative, room-temperature etching method using halogens in anhydrous solvents to synthesize MXenes from Ti_3_AlC_2_, induces the generation of –Br and –I terminations.^23^ More recently, a one-step elemental tellurium etching strategy has been demonstrated for a range of MAX precursors (Ti_3_AlC_2_, V_2_AlC, Nb_2_AlC, Ti_2_AlC), for which Te acts simultaneously as the selective etchant and the terminating species, yielding Te-terminated MXenes without requiring any fluoride reagents.^24^

### Post-synthesis termination engineering

Even after synthesis, the termination chemistry can be substantially modified through posttreatment methods, enabling access to compositions not directly achievable during etching. Thermal annealing in vacuum is one of the most widely used approaches since –OH terminations are removed above approximately 350°C, while –F desorbs as F_2_ gas above ~500°C, leaving behind –O as the sole, thermally persistent termination.^1,14,22^ After the removal of –OH and –F terminations, full –O coverage can be obtained by controlled exposure to O_2_ gas at temperatures up to 100°C. However, higher temperatures (above 400°C) or prolonged exposure can induce oxygen supersaturation and eventually lead to irreversible MXene degradation, forming TiO_2_, CO_2_, and desorbed H_2_O as reaction products.^25^ Crucially, Hart et al. demonstrated that this –O enrichment is reversible. Plasma annealing in an Ar + O_2_ atmosphere after vacuum defunctionalization refunctionalizes the surface with –O, while minimizing secondary TiO_2_phase formation, enabling an iterative and controllable toggling of the –O content.^14^

Another route to remove –F termination involves hydrolyzation by gas-phase H_2_O. A continuous gas flow of humidified Ar(g) has been successfully employed to produce nearly F-free and partially bare multilayered V_2_CT_x_, which was also confirmed as more thermodynamically stable by theoretical calculations.^26^ The use of a continuous gas flow offers a important improvement to previously reported hydrolysis methods in a closed environment, for which the buildup of partial pressure of product gases rapidly hinders the reaction. The choice of the temperature is crucial also for this method, with 300°C being the optimal value to remove –F terminations, while temperatures above 400°C result in the decomposition of the MXene into V_2_O_3_ and C.^26^

**Figure 3:**
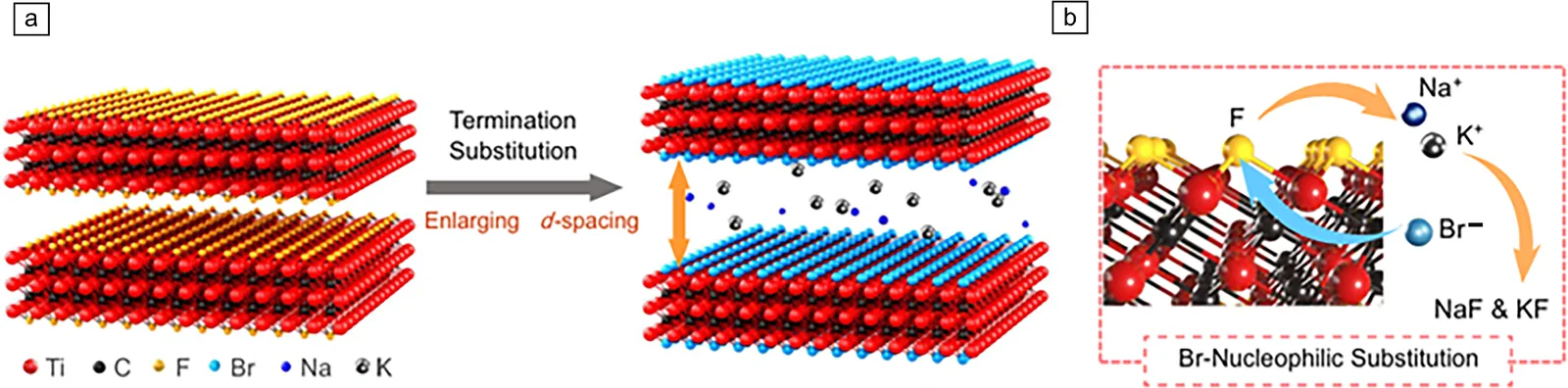
Liquid phase post*-*synthesis termination substitution. Treatment with Lewis*-*basic halide salts can substitute –F terminations with halide anions (such as Br–) and simultaneously enlarge interlayer spacing by intercalating alkali cations. Reprinted with permission from Reference 28.

Liquid phase treatments offer an alternative post-processing strategy to control MXenes’ terminations. Alkaline treatment with KOH or NaOH replaces –F by –OH through the formation of soluble NaF or KF salts, and subsequent annealing converts –OH to –O, providing a two-step route to predominantly oxygen-terminated MXenes.^27^ The treatment with Lewis-basic halide salts (e.g., AlBr_3_NaBr/KBr eutectic melts) substitutes native –F by nucleophilic reaction with desolvated halide anions, simultaneously enlarging the interlayer spacing via intercalation of alkali cations,^28^ as visible in **Figure** **3**.

Electrochemical post-synthesis treatments have also been studied to modulate *in* *situ* the surface terminations of Ti_3_C_2_T_x_. A cycled variation of the applied external potential reversibly enabled the *in* *situ* switching between –O- and –OH-rich surfaces via protonation/deprotonation of terminations, without affecting the –F fraction.^29^

## Environmental stability of MXenes in solution and in deposited films

Despite their promising properties, MXenes suffer from a critical practical limitation: they can easily undergo degradation through hydrolysis (upon exposure to ambient conditions) or through oxidation (at high temperatures). These processes result in the formation of TiO_2_ and carbon by-products, associated with loss of metallic conductivity and of the original layered structure. Understanding the factors that govern degradation and how they are influenced by surface terminations is essential for any application requiring long-term material stability, whether in colloidal dispersions or solid films.

### Degradation mechanisms

#### Role of water, oxygen, and surface chemistry

MXenes are known to be particularly susceptible to degradation in colloidal solutions. However, a comprehensive understanding of the oxidation mechanisms and key factors that affect them has yet to emerge. Early studies^30^ identified dissolved O_2_ as the primary oxidant of MXene dispersions, thus degrading MXenes’ layered structure to form TiO_2_anatase and amorphous carbon.^31^ A conceptual shift came from Huang et al., who demonstrated that water is the dominant degrading agent instead of O_2_.^32^ They systematically compared Ti_3_C_2_T_x_ and Ti_2_CT_x_ in aqueous and nonaqueous colloids exposed to oxygen and inert gas atmospheres, observing a notably faster oxidation rate in oxygen-free water solution compared to nonaqueous environments saturated with oxygen. A follow-up study extended to Ti_3_CNT_x_ observed that the degradation product (besides TiO_2_ nanoparticles) in water was mainly CH_4_ (and NH_3_ for Ti_3_CNT_x_), thus confirming that Ti–C/Ti–N bond hydrolysis by water is the primary reaction pathway.^33^ A more recent study confirmed the presence of additional minor products (acetylene, ethylene, and ethane) arising from secondary radical recombination, and of growing fractions of carbon oxides in environments with increasing oxidation potentials (air, O_2_, and H_2_O_2_).^34^ Following from these results, the storage lifetime has been successfully improved by using water- and oxygen-free environments, such as dimethyl sulfoxide (DMSO) and dimethylformamide (DMF).^35^ However, this approach still presents challenges due to the low solubility of MXenes containing –OH, –O, and –F surface terminations in nonpolar organic solvents.^35^

**Figure 4:**
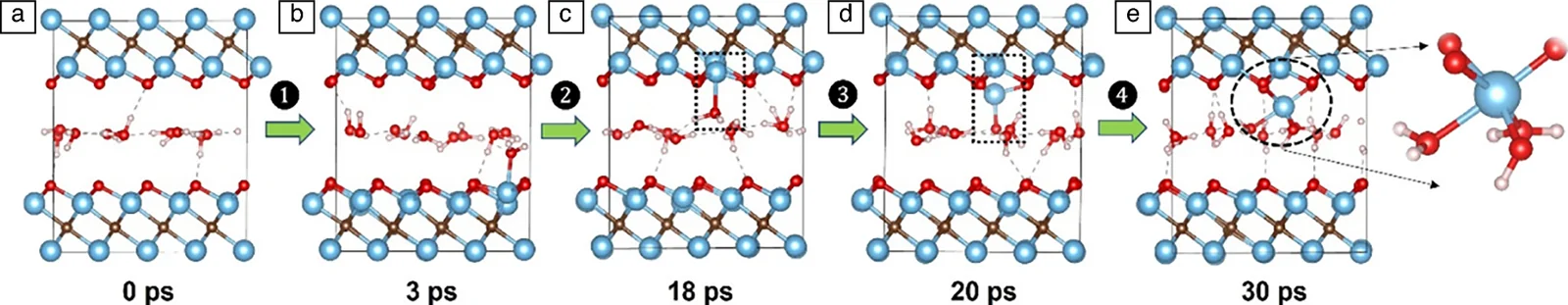
Atomistic insights on the first stages of water attack, that eventually lead to hydrolysis and degradation of the MXene structure, obtained with *ab* *initio* molecular dynamics simulations. The process is composed of several stages: after an initial phase of reversible adsorption on Ti, water chemisorbs irreversibly on a Ti atom and later dissociates, causing Ti pullout, exposing the Ti to further reaction with neighboring water molecules. Reprinted with permission from Reference 36. © 2022 American Chemical Society.

The atomistic mechanism of the water attack has been elucidated by a convergent body of simulation work. First-principles molecular dynamics (MD) on Ti_3_C_2_O_2_ (**Figure** **4**) showed that water adsorbs reversibly on superficial Ti atoms before irreversibly chemisorbing, dissociating, and physically pulling the Ti atom out of the basal plane via nucleophilic attack on the electron-deficient metal center, breaking Ti–C bonds.^36^ Further simulations on V_2_CO_2_ confirmed the generality of this mechanism and additionally showed that nonpolar O_2_ cannot attack metal atoms while they are still protected by surface functional groups, but can do so once a metal atom is already protruding from the surface, explaining why O_2_ acts as a secondary rather than primary oxidative agent.^37^ Additional *ab* *initio* MD simulations by Nesterova et al.^38^confirmed that the susceptibility of a given MXene surface to water attack is highly sensitive to the local termination chemistry, with the activation energy for Ti removal spanning an order of magnitude (from 0.28 eV on an O-terminated surface to more than 3.9 eV on an OH-terminated one). This underlines that the termination composition is a primary determinant of MXenes’ kinetic stability. Several paths to reduce oxidative degradation have emerged from simulations. For instance, negatively charging the surface can repel the oxygen of water molecules, preventing the adsorption through Ti–O bonds that eventually lead to oxidation. Alternatively, substituting –O terminations with –OH groups can physically prevent close water–Ti encounters and prevent water attack,^36^ which is directly relevant to termination-based passivation strategies. A different study assessing the thermodynamic stability in aqueous solution of a wide range of MXenes with different metal and carbide/nitride compositions identified –O termination as the most thermodynamically stable among mixed –F/–OH/–O terminations produced by HF-based synthesis.^39^ On the other hand, the –O termination also possesses the lowest activation barrier for Ti removal,^38^ suggesting a nontrivial interplay between thermodynamic stability and kinetic stability to oxidation.

#### Role of structural defects, flake size, and processing quality

Experimentally, degradation initiates at the most reactive sites on the MXene surface, which relate to undercoordinated Ti atoms at flake edges and at vacancy defects in the basal plane, from which TiO_2_ nucleates and spreads laterally.^39–41^ *Ab* *initio* calculations confirm this picture quantitatively since the water chemisorption energies are 0.60–1.80 eV at vacancies and edge sites, compared to mere physical adsorption on defect-free basal planes.^42^ Defects expand autocatalytically once formed, as incoming water molecules preferentially attack the already undercoordinated Ti atoms surrounding a preexisting vacancy.^37^

Smaller flakes, which have a higher edge-to-surface ratio, therefore degrade considerably faster than larger ones, while higher-quality MAX precursors with fewer inherited defects yield MXenes with a superior colloidal stability.^43^ Reducing HF concentrations lowers the amount of Ti vacancies,^44^ while using an excess Al in the parental MAX phase helps to generate a more stoichiometric and crystalline product with improved stability.^45^ Moreover, pH further modulates kinetics since acidic conditions catalyze degradation by protonating surface –OH groups and increasing electron localization on Ti, while moderately basic conditions partially suppress oxidation.^46^

#### Stability of deposited films

With respect to deposited films, interlayer stacking limits the accessibility of water and O_2_to inner layers, making well-aligned, dense films considerably more resistant to bulk oxidation than colloidal dispersions.^41^ A multiyear study on Ti_3_C_2_T_x_ freestanding films aged for about 4 to 10 years verified that the primary cause of conductivity loss under ambient storage is reversible water uptake between the hydrophilic MXene layers (and not irreversible oxidation). In this regard, vacuum annealing was able to partially or fully recover the initial conductivity for many samples.^47^ Films processed with HF/H_2_SO_4_, yielding clean surfaces free of Cl terminations and residual Al salts, retained approximately 90% of their initial conductivity after five years of laboratory storage. This confirmed that minimizing the amount of reactive surface species and reducing the interlayer spacing (thereby preventing water and air from entering) are the decisive factors for long-term film stability. Moreover, methods such as hydrogen annealing have been proposed to improve the resulting oxidative stability and even partially recover electrical conductivity of solid Ti_3_C_2_T_x_ films.^48^

Concerning the high-temperature stability of MXenes, direct oxidation by O_2_ is the main degradation pathway. The role of oxygen at high temperature was elucidated by Li et al., who showed that Ti_3_C_2_T_x_ powder with F/OH terminations is stable in argon atmosphere up to 800°C, while in oxygen atmosphere oxidation starts above 200°C to form anatase nanocrystals, which subsequently transition to rutile at higher temperatures or longer exposure times.^49^

#### Passivation strategies: Surface and edge functionalization

As the degradation process is mainly initiated at undercoordinated Ti at edges and defect sites, several oxidation prevention strategies have been attempted to passivate these reactive sites through covalent or noncovalent surface and edge functionalization.

The Lewis acid character of undercoordinated Ti atoms in edges and vacancies makes them natural targets for Lewis base ligands. Yun et al.^50^ verified this approach by functionalizing Ti_3_C_2_T_x_ with pyrrole, an aromatic amine, achieving structural integrity for more than 700 days, compared to just 60 days of pristine MXene. Extending this concept, N-containing ligands with long alkyl chains, such as oleylamine, simultaneously passivate the Ti sites via covalent N–Ti bonding and at the same time make the surface hydrophobic due to the long alkyl chains, reducing contact with water.^51^

A complementary route is constituted by phosphonic acids. In this regard, the phosphonate group reacts with metal-coordinated –OH groups to form stable Ti–O–P bonds, and the long hydrophobic alkyl chain then creates a water-repelling barrier.^52^

**Figure 5:**
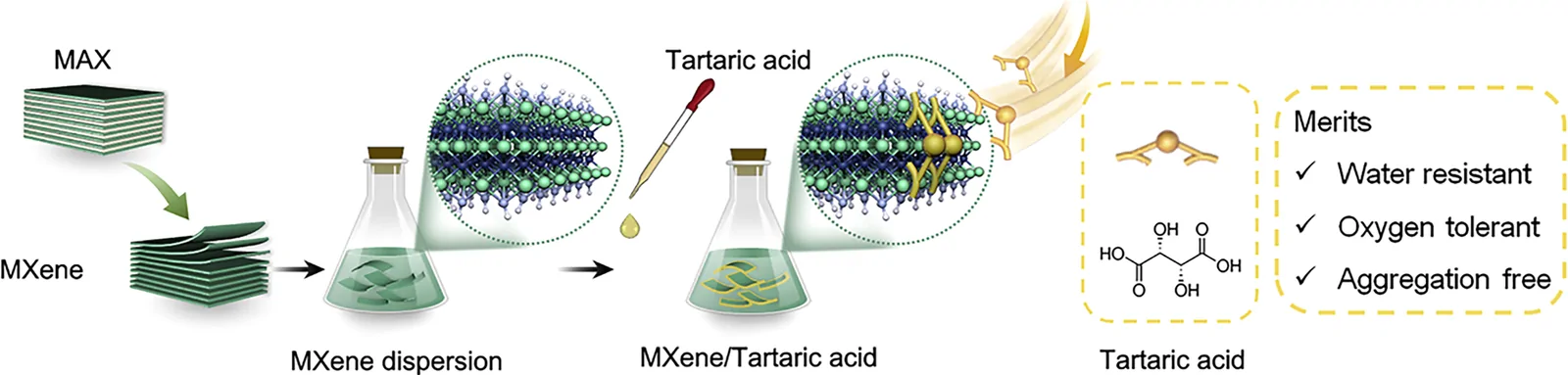
Edge capping method to prevent degradation in water solution by means of tartaric acid. By bonding at undercoordinated edge sites, it prevents water attacks that can initiate at the edges and propagate through the basal plane, effectively protecting the MXene from hydrolysis. Reprinted with permission from Reference 53.

Among capping agents, tartaric acid has also achieved a notable oxidation reduction at 60°C in water solution. It works by bonding to under-coordinated edge sites, effectively protecting defective edge Ti sites from water attack that can initiate oxidative degradation that propagates from the edge through the MXene plane,^53^ as sketched in **Figure** **5**. Moreover, it also improves the stability of composite structures, such as Ti_3_C_2_T_x_/PEDOT:PSS (i.e., poly(3,4-ethylenedioxythiophene) poly(styrene sulfonate)), and can increase their electronic conductivity.^53^

For MXenes rich of –OH surface termination, an efficient functionalization method relates to hydrolysis reactions with silanes, eventually forming covalent Ti–O–Si bonds. Organosilane coupling agents (e.g., (3-aminopropyl)triethoxysilane—APTES) provide covalent Ti–O–Si bonds with an additional protective functionality from the terminal group,^54^ whereas fluoroalkyl silanes have been used to create superhydrophobic surfaces.^55^

The positively charged character of flakes edges and defects renders them susceptible to electrostatic adsorption of anionic species, which can be exploited for passivation. Polyanionic inorganic salts (polyphosphates, polysilicates) tend to bind to edge Ti sites and create an oxidation-resistant blocking layer without affecting the electronically conducting basal plane.^56^ Anionic salts containing long alkyl chains, such as sodium dodecyl sulfate (SDS), adsorb on both surface and edges, improving the resulting hydrophobicity and extending the colloidal shelf life to over 200 days.^57^

For deposited films, polymer coatings offer a practical route for passivation that simultaneously improves the film microstructure. Polydopamine (PDA) is particularly noteworthy since it undergoes spontaneous *in* *situ*polymerization and binding at the flake surfaces when added to MXene dispersions prior to vacuum filtration. This helps to produce ultrathin adhesive layers that improve interflake ordering, adhesion, and resistance to oxygen and moisture.^48^ More broadly, both synthetic polymers and biopolymers (e.g., silk fibroin^58^) can coordinate with MXenes’ surface terminations through a combination of covalent bonds, hydrogen bonding, and van der Waals interactions, forming a hydrophobic shell that repels water and O_2_ while preserving the intrinsic 2D structure (**Table** **I**).

**Table I:** Summary of mechanisms, stability improvement, additional benefits, and limitation of various passivation strategies.

Agent(s)	Target Site/Mechanism	Stability Gain	Added Benefit	Limitation
Lewis base ligand**:** heterocyclic aromatic amines (pyrrole)^50^	Coordination to Lewis-acidic undercoordinated Ti at edges/vacancies; forms strong TiC bonds	>700 days versus 1 month in water at room temperature; 42 days versus 1 day in water at 70°C	Enhances electrochemical performance as a supercapacitor	Edge/defect-selective
N-ligand + alkyl chai**n:** Oleylamine (OAm)^51^	Covalent Ti–N bonds and H-bonding; develops an ordered hydrophobic alkyl monolayer	Stable for 30 days in chloroform versus <10 days for pristine in water	Enables transfer to nonpolar solvents; improved spray-coating performance	Decreased electrical conductivity due to intercalation of insulating ligands
Alkylphosphonic acids (CnPA)^52^	Covalent Ti–O-P tridentate bonds with surface –OH sites via interfacial condensation	Retains single-sheet morphology and crystallinity for 3 months in chloroform	Allows dispersion in various organic solvents, from highly polar to nonpolar	Requires –OH-rich surfaces; less effective on –O or –F terminated MXenes
Tartaric acid^53^	Caps undercoordinated edge Ti sites; provides a reducing effect	Significant oxidation reduction in water even at 60°C for at least 2 weeks	Improved structural stability and conductivity in MXene/PEDOT:PSS composites	Edge-selective
Organosilane**:** APTES^59^	Silylation reaction with surface –OH groups to from Ti–O–Si bonds	Degradation reduced to 20.8% after 11 days (versus 95.6% for pristine)	Adjustable hydrophilicity, suppressed nanosheet restacking	High concentrations can negatively impact metallic conductivity
Fluoroalkyl silane**:** FOTS^55^	Covalent Ti–O–Si bonds; long fluoroalkyl chains form a superhydrophobic protection layer	Maintains 86.2% conductance after 2 weeks in 100% relative humidity	Enhanced gas-sensing performance	The insulating layer may reduce conductivity
Polyanionic salt**s:** polyphosphates, polysilicates, etc.^56^	Electrostatic adsorption/capping of positively charged edge sites	Significantly slows oxidation in aerated water for >3 weeks	Salts can be easily washed away	Edge-selective
Anionic surfactant**:** Sodium dodecyl sulfate** (**SDS**)** ^57^	Sulfate group binds to defects via Coulomb attraction; alkyl tails repel water	Extended colloidal stability for up to 213 days	Negligible influence on capacitive properties after rinsing	Noncovalent; excessive SDS concentration causes micelle repulsion and instability
Polymer coating**:** Polydopamine (PDA)^48^	Spontaneous *in situ* polymerization and binding at flake surfaces	Enhanced resistance to oxygen and moisture during long-term ambient storage	Improves interflake ordering, adhesion, and mechanical strength	Film-specific strategy, not for colloidal dispersion
Biopolymer coating**:** Silk fibroin^58^	Encapsulation via dense hydrophobic sheets	Extended storage life in aqueous environments	Biocompatibility	Encapsulation reduces bulk conductivity in membranes

## MXenes stability in tribological conditions

**Figure 6:**
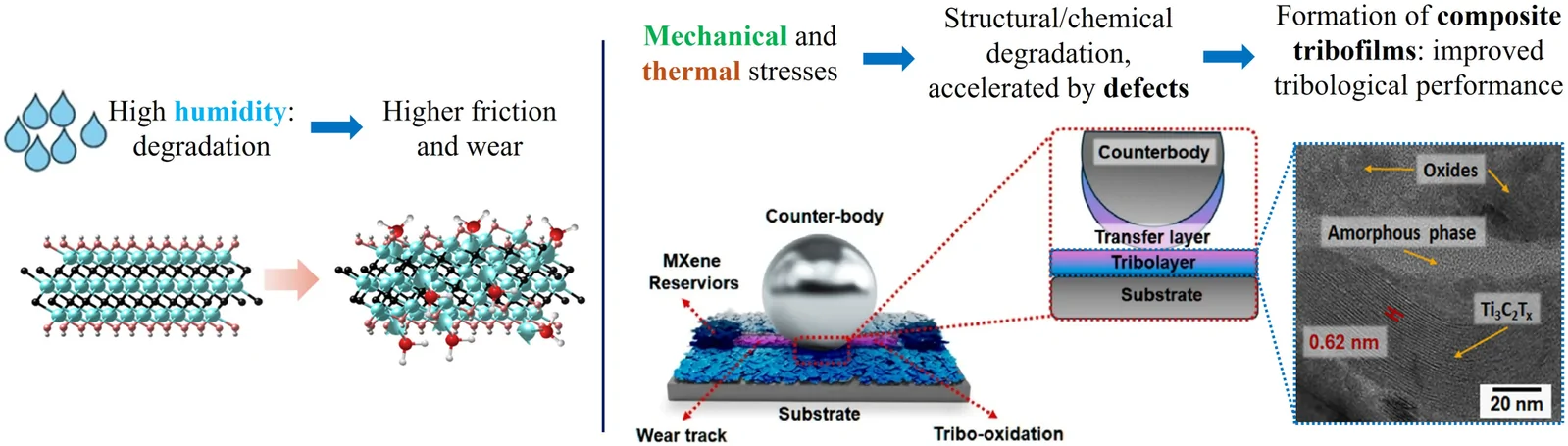
Overview of the effects of humidity, mechanical and thermal stresses induced by load and shear on the tribological performance of MXenes. Adapted with permission from Reference 62. Adapted with permission from Reference 63. © 2025 American Chemical Society. Adapted with permission from Reference 64. © 2025 Elsevier.

As outlined before, MXene flakes are prone to oxidization and degradation under certain environmental conditions including ambient conditions and –OH containing environments. Related to the latter, these environments can include air exposure with elevated relative humidity levels, water or aqueous solvents.^60,61^ Under these conditions, the enhanced reactivity of MXene surfaces due to their early transition metal, the existing surface terminations induced by chemical wet etching as well as generated defects (M-, X-, and T_*x*_-vacancies/clusters or combinations) induces and accelerates the involved degradation processes (**Figure** **6**).

Under tribological testing, these degradation processes can be further amplified and accelerated by several contributions. In this regard, tribological experiments should be conducted adjusting the respective temperature and relative humidity. Both aspects can play a crucial role regarding the corresponding friction and wear performance of MXene coatings.^12,65^ Regarding temperature, it is well-accepted in literature that with increasing environmental temperature, MXene flakes undergo certain chemical and/or structural changes. When considering a minor temperature increase under ambient conditions (temperature up to 150°C), the existing surface terminations (starting with –F terminations and moving toward –O and –OH groups) become released.^66^ Modifications of the respective surface chemistry by removing surface termination may not be decisive for micro-/macroscale tribology when applying contact pressures on the order of several hundreds of megapascals. However, it can induce important and measurable effects for nanotribological studies, which make use of applied normal loads on the order of nN.^12,65^ To the best of our knowledge, the effect of specific surface terminations on the resulting nanoscale friction and wear performance has not been fully understood and explored. This aspect largely connects with the fact that MXene flakes typically contain mixed surface terminations. As outlined above, from a synthetic point of view, it is not straightforward to generate homogeneous and uniform one-type surface terminations. In this regard, advances in synthetic routes and post-synthesis approaches may help to shed more light on this rather unexplored topic.

Due to the involved and up to now state-of-the-art synthetic approach based on wet-chemical etching, most MXenes contain a certain amount of intercalated water.^66^ This water can be absorbed to the top layer of the flakes or intercalated in between MXene layers. When increasing the environmental temperature from room temperature up to 100–150°C, it has been reported that first physically absorbed water can be removed from the surface, while intercalated water becomes removed for temperature around 200–300°C^.66^ From a nano-, micro- and macroscale tribology point of view, it still remains unclear whether the existence of intercalated water is beneficial or detrimental for the resulting friction and wear performance.

When further increasing the environmental temperature and exceeding the onset of oxidation, MXene flakes start to chemically degrade/oxidize. These oxidation processes start at the surface, with a higher probability to occur at defect accumulations or edges. Oxidation processes initially lead to superficial oxidation while maintaining the original MXene core. For elevated exposure times or further increased temperatures, oxidation proceeds toward the bulk of the flakes thus transforming the MXene structure into a layered oxide structure (e.g., Ti_3_C_2_T_x_ transforms into layered TiO_2_).^67^ Depending on the considered MXene type (early transition metal), it depends on the formed oxidic species, whether these species can help to induce a beneficial friction and wear performance.

Concerning the relative humidity, regardless of the respective scale of the tribological experiments, low relative humidities are favorable due to the ongoing degradation processes induced by –OH. This aspect has been experimentally confirmed by Marian et al., who generated multilayer Ti_3_C_2_T_x_ coatings and tested them macroscopically under humidity levels of 20 and 80 percent. In this regard, Marian et al. verified a low and stable friction evolution for a relative humidity of 20 percent. In contrast, a detrimental tribological performance was observed for humidity levels around 80%, which was traced back to the induced swelling and structural/chemical degradation of Ti_3_C_2_T_x_under these conditions.^60^ These results align well with another experimental study conducted by Boidi et al., who confirmed similar effects for hybrid coatings based on Ti_3_C_2_T_x_ and MoS_2_.^61^

Apart from the adjusted temperature and relative humidity, the applied normal load and resulting contact pressure must be taken into consideration. In this regard, it is important to emphasize that tribological rubbing always induces a superimposed mechanical and thermal stress to the sample tested. The applied Hertzian contact pressure locally increases by several orders of magnitude due to the involved roughness scale, which induces a local temperature increase. This combined thermomechanical impact certainly affects the stability of MXene flakes and structural integrity of MXene coatings.^12,65,68^

Based on the existing state of the art, it is well accepted that MXenes’ friction and wear performance is highly connected with formation of beneficial tribofilms induced by tribochemistry. In this regard, the as-deposited MXene coating becomes thinner as well as structurally and chemically degraded. This implies that some of the initial coating material is worn off and/or transferred to the tribological counterbody. Moreover, the initial MXene flakes start to break and reduce their *x*- and *y*-dimensions. Due to the involved rubbing and shear motion, MXene flakes become exfoliated thus reducing their *z*-size and potentially transforming multilayer stacks into few- or even monolayer flakes. From a chemical point of view, MXene flakes tend to oxidize under the respective thermal–mechanical impact. These structurally and chemically degraded flakes become intermixed with original MXene flakes and oxide species originating from the substrate thus forming a composite tribofilm on the substrate. This tribofilm can be further transferred to the rubbing counterbody to establish a tribofilm/tribofilm interface, which accommodates all the energy-dissipating processes. When considering all these processes, which lead to the formation of these beneficial tribofilms, it becomes evident that MXenes’ reactivity and the involved thermomechanical impact are beneficial and needed to some extent. In this regard, it can be envisioned that MXenes’ reactivity can be used to tailor, fine-tune, and manipulate the involved tribofilm formation. By intentionally increasing the respective reactivity (for instance by an increased defect density) in a local manner, it appears to be possible to locally initiate and guide the tribofilm formation.^12,65,68^

Apart from the overall reactivity, the friction and wear performance as well as the overall stability of the films and induced performance depend on the respective coating-substrate adhesion.^12,65,69^ On the one hand, the adhesion to the underlying substrate must be sufficient to avoid the coating removal/peel-off. On the other hand, adhesion should not be too strong to enable a certain material transfer to the acting counterbody. This implies that intermediate adhesional energies of the MXene flakes with respect to the substrate and counterbody material seem to be the best compromise.

## Role of surface terminations in the tribological performance of MXenes

**Figure 7:**
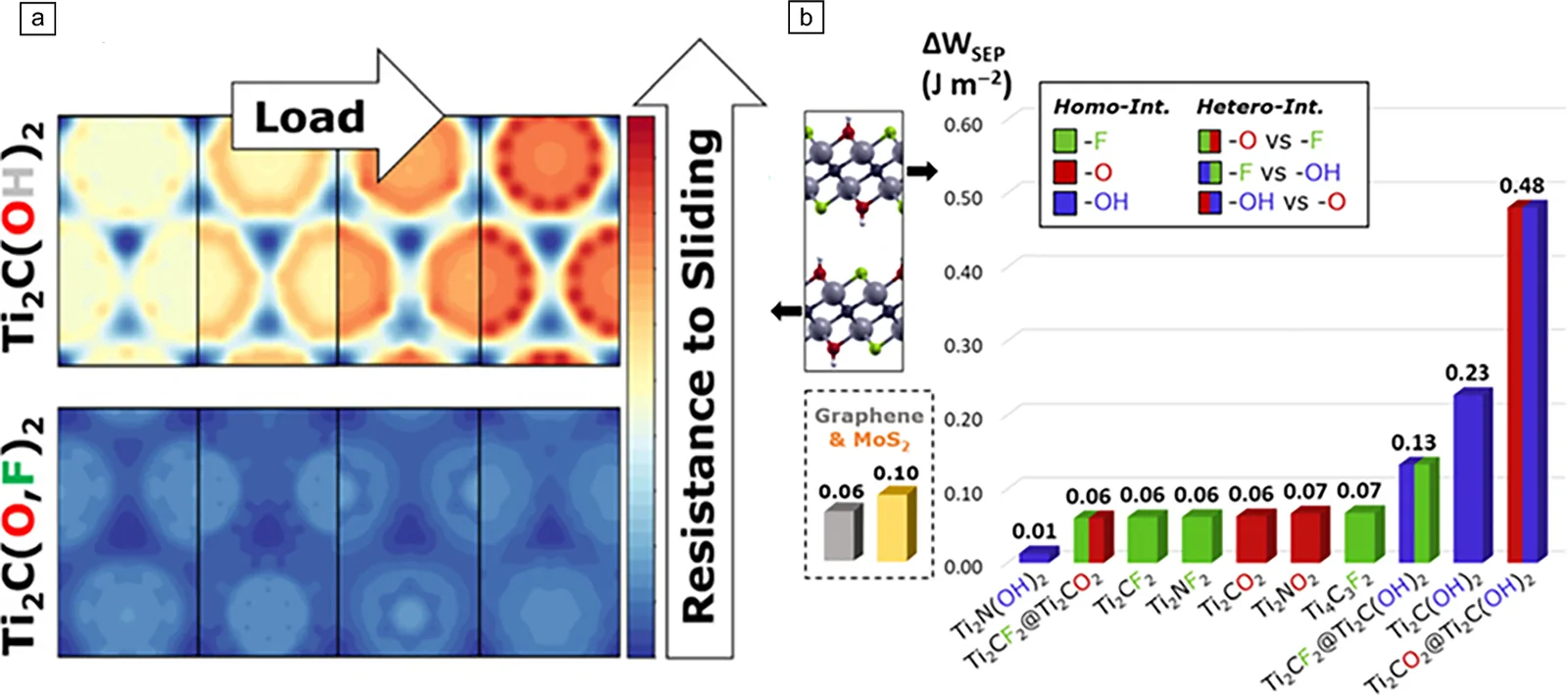
Effect of surface terminations on the nanotribological behavior of MXenes. From *ab initio* calculations, –OH terminations exhibit higher corrugation in the potential energy surfaces (a) and increased adhesion (b) compared to –O and –F, translating to higher resistance to sliding. Adapted with permission from Reference 70.

When aiming at assessing the effect of the existing surface terminations on the resulting tribological performance, it is essential to distinguish between the behavior occurring at different scales (nano- versus micro-/macrotribology) (**Figure** **7**).

In case of the nanotribological behavior, the acting intermolecular forces between adjacent MXene layers are roughly on the same order as the applied normal forces. This implies that the involved surface chemistry and existing surface terminations notably influence and tailor the resulting frictional performance on this length scale. From a theoretical point of view, Marquis et al. verified that the resulting adhesional strength of MXene layers to the underlying substrate (iron and iron oxide) is predominantly governed by the surface terminations. They demonstrated that the most favorable frictional performance is induced by homogeneously distributed –O terminations.^70^ Zhang et al. theoretically predicted that MXenes without surface terminations tend to have an increased frictional resistance due to the generation of metallic bonds.^71^ Similarly, Hu et al. revealed that the existing functional groups generally weaken the interlayer coupling, which can help to manipulate the resulting frictional performance when solely replying on interlayer slidin^72^ Using DFT calculations and MD simulations with ReaxFF potentials, Zhang et al. investigated the friction of adjacent layers of For Ti_n+1_C_n_ with n ranging from 1 to 3, Zhang et al. demonstrated the certain type of surface terminations such as –OH and –OCH_3_notably reduce the frictional resistance. Moreover, they theoretically underlined the important effect of etching-induced defects regarding the resulting reactivity, nanoscale roughness, and interlayer attraction.^73^ From an experimental point of view, Guan et al. synthesized Ti-based MXenes with –O and –OH as well as –F, –Cl, and –Li terminations thus verifying the notable impact of the surface terminations on the resulting nanotribological performance.^74^ Serles et al. demonstrated that the manipulation of MXenes’ surface terminations by thermal annealing, partially replacing –OH terminations with –O and –F, can help to reduce friction at the nanoscale by up to 57 percent.^75^ Although these initial studies show some pronounced effects of the surface terminations on adhesion and friction at the nanoscale, additional systematic research work is necessary to explore their full effect on the nanotribological performance. In this regard, theoretical work and predictions should go hand in hand with experimental effort, which must start with an optimized synthetic route to create homogenous surface terminations on MXenes flakes. This is of utmost importance because the nanotribological response is expected to be majorly affected by local heterogeneities and variations in the respective chemical species.

While there is a direct effect of the surface terminations on the tribological performance at the nanoscale, the resulting friction and wear response is indirectly affected on the micro- and macroscale. For micro- and macroscale tribological, the applied forces and resulting contact pressures are orders of magnitude larger than the intermolecular forces. However, the existing surface terminations of MXenes play a major role for their chemical functionalization, which is highly beneficial to boost their performance as lubricant additive and solid lubricant coatings.^59^ Due to their inherent hydrophilicity, MXene flakes do not have a good chemical compatibility with apolar liquids such as oil and greases. This typically induces a pronounced phase separation and downgraded dispersion, which is highly detrimental for the stable and safe operation of lubricants. In this regard, chemical functionalization is an excellent approach to make MXenes more hydrophobic and, therefore, better compatible with base oils and greases.^59^

In this context, different chemical species and bridging molecules can be used to intentionally tune and tailor MXenes’ surface chemistry. Particularly, silane functionalization of MXenes has emerged as an effective strategy to enhance its surface properties, stability, and tribological behavior. Moreover, dialkyl dithiophosphate (DDP) functionalized Ti_3_C_2_T_x_ has been explored as a lubricant additive to enhance the resulting tribological response. Gao et al. verified that the functionalized MXenes demonstrated an improved dispersion stability and reduced aggregation, and improved tribological properties in base oil were verified. Tribo-testing showed that DDP functionalization prevented agglomeration and enabled a stable tribofilm, which notably decreased friction and wear.^76^ The chemical functionalization with organic acids points toward a similar direction thus improving their surface stability, dispersion, and mechanical properties. Liu et al. coupled Ti_3_C_2_T_x_ with zinc dialkyldithiophosphate (ZDDP) by a straightforward surface grafting approach. In this regard, MXenes were chemically grafted with ZDDP moieties, which enabled their homogeneous dispersion in pure base oils and boosted their chemical affinity to the underlying substrate. By this approach, more stable and robust tribofilms were formed, which helped to reduced friction and wear by 50 and 60%, respectively, when compared to tribotesting with oils containing MXenes without ZDDP functionalization.^77^ Although several successful experimental studies on chemical functionalization of MXenes and their usage as lubricant additives are available,^78,78,80^ more systematic studies are currently missing. In this regard, it would be appreciated to receive input from high-throughput simulations to select the respective functionalization approach, which should be tailor-made for the lubricant considered and underlying surface chemistry of the interacting materials. We anticipate that this is an important step to reduce the cost-intensive and time-consuming trial-and-error approach thus aiming toward a resource-efficient design of lubricant additives.

When thinking about MXenes’ application and usage as solid lubricant coatings, a baseline and benchmark comparison with other established 2D systems helps to shed light on MXenes’ overall potential. Traditional solid lubricant coatings based on 2D materials are commonly made of graphite, graphene, and MoS_2_.^81,81,83^ In this regard, the mentioned materials have shown great success in solid lubrication under specific testing conditions. Concerning MoS_2_, it is well established that this material system functions best under vacuum, dry and low-humidity conditions thus creating low-friction tribofilms and making it the material of choice for aerospace applications.^83^ In case of graphite or graphene, these materials work best under more humid conditions thus inducing ultralow frictional states.^84,84,86^ Consequently, each 2D material system comes with its own system- and environment-depending advantages and bottlenecks, which implies that there is no 2D material that works as a solid lubricant across the entire humidity range. Moreover, while these systems show an excellent frictional performance with ultralow COFs, their durability and wear resistance are two limiting factors. Once the first signs of wear initiate, the systems using graphene, graphite, and MoS_2_ tend to fail catastrophically over time. To overcome these drawbacks, notable work has been dedicated toward the design of hybrid coating systems combining graphene with MoS_2_.^87,87,89^ Although shown beneficial synergetic effects, the greatest bottleneck related to durability and wear resistance has not been overcome by this approach. In this context, MXenes come into play due to their ceramic-like nature originating from their MAX phase precursors. Unifying good mechanical properties/stability with a tunable surface chemistry, enhanced surface reactivity, and lamellar 2D structure, the usage of MXenes as solid lubricant coatings has been one of the greatest success stories until now. Although generally not offering ultralow friction, MXene coatings tend to form wear-resistant, durable tribofilms on the underlying substrate, which is then transferred to the tribological counterface.^68^ Considering the almost infinite design space regarding the M, X, and T_x_ chemistry together with the excellent results on their solid lubrication ability, it can be envisioned that MXenes’ solid lubrication performance can be further optimized and improved. In this regard, the existing surface terminations undoubtedly manipulate and determine the interaction and adhesion with the underlying substrate. Marquis et al. demonstrate in a recent study that the substrate chemistry is decisive for the adhesional strength and interaction with MXenes’ terminal groups. The resulting adhesional strength is also crucial for the establishment of stable tribofilms, which are supposed to stick to the underlying substrates. Although of major importance, little theoretical and experimental insights are available, which paves the way for more research activities in this promising direction. Considering that solid lubrication is one of the key topics in MXene tribology due to the extended wear resistance and durability, more research in this direction is expected to further boost the resulting friction and wear performance. Based on theoretical predictions, educated guesses should be derived with the overall intention to optimize the resulting adhesional strength by adjusting the chemical functionalization approach with regard to the interfacial characteristics of the tribosystems. In this regard, the adhesional strength should be sufficient to avoid coating removal, but still low enough to enable the transformation of the MXene coatings into beneficial tribofilms.

## Summary and outlook

Based on this review, the important role of MXenes’ surface terminations on stability and the resulting tribological performance has been critically discussed. Starting with the different options for chemical wet etching to transform the parental MAX phases into MXenes as well as the respective implications for the corresponding surface chemistry/terminations, we have dived into the environmental stability of MXenes in solution and as deposited films. The crucial interplay between –OH containing environments (water, ambient conditions, solvents) and MXenes’ surface chemistry has been elucidated, while also the effects of defects and flake size have been considered. To enhance the environmental stability of MXenes in dispersion or as coatings, surface functionalization has been identified as an excellent approach to fine-tune surface chemistry and reactivity.

Apart from that, MXenes’ stability under tribological conditions has been reported. In this regard, it is important to emphasize that MXenes’ reactivity with the tribological environment is the key aspect for the involved mechano-tribochemistry and formation of beneficial tribofilms. The partial structural and chemical degradation under rubbing actions due to the involved temperature and mechanical stress transform the initial MXene flakes/coatings into a highly densified, compacted reaction layer (tribofilm). This layer consists of pristine MXene, structurally and chemically degraded MXene flakes, and different oxide species thus resembling a composite tribofilm.

Regarding the role of MXenes’ surface terminations on the resulting friction and wear performance, the literature landscape is rather scarce. Although some theoretical studies have tried to assess the effect of surface terminations on friction, a systematic experimental verification is still missing. Some experimental knowledge is available based on nanotribological measurements, but the influence and effect of their terminations on the micro- and macroscale tribological performance are rather unexplored.

To shed more light on the potential effects of MXenes’ surface terminations, several key aspects can be considered. In this regard, MXenes’ synthesis and post-synthetic treatments need to be improved to reliably generate MXenes with preferential surface terminations, which should be homogeneously covered and distributed over the entire flake surfaces. After careful characterization, this would be the key prerequisite to allow nanotribological studies assessing the effect on friction and nano-wear (edge wear) considering specific types of surface terminations. These experiments can be coupled with *in situ* or *operando* characterization techniques such as infrared and Raman spectroscopy to elucidate the dynamic evolution of the involved surface chemistry upon tribological impact. After understanding the effects on the nanoscale using friction force microscopy and nanotribological experiments, the experiments should move to coarser scale involving micro- and macrotribological experiments.

Regarding the MXene chemical composition, to date only Ti-based carbide systems constitute the state of the art of MXene tribological studies across different scales. Considering that MXenes comprise an entire family of carbides, nitrides, and carbonitrides with a notable diversity in chemistry, structure, and stoichiometry, it can be concluded that the overall topic of MXene tribology is still in its infancy. Combined efforts making use of theoretical predictions and experimental validation should be bundled to shed more light on the tribological performance of this promising class of materials. High-throughput DFT simulations should be used to calculate adhesional energies and slipperiness with different substrates considering the available early transition-metal chemistry, wealth of surface terminations and stoichiometries. These predictions should be cross-correlated and validated with nanotribological experiments. These results can be used as input parameters for more advanced MD simulations, which can be coupled with ML algorithms to predict tribofilm formation and the underlying kinetics as well as pathways depending on the MXene variables (chemistry, structure, and stoichiometry). Based on these predictions and experiments, the existing know-how in MXene tribology can be extended toward other transition-metal chemistry.

Considering the important influence of MXenes’ overall reactivity on the tribofilm formation of Ti-based MXene (single-metal MXene), the community has recently explored the possibility of engineering specific defects in MXenes’ structure. These defects have been theoretically shown to enhance their overall surface reactivity, which may be a promising approach to accelerate the associated chemistry in tribological systems. Apart from single-metal MXenes, the MXene community has moved toward the investigation of ordered double transition-metal MXenes such as Mo_2_TiC_2_ or Mo_2_Ti_2_C_3_, which are almost fully unexplored from a tribological point of view. These MXene structures add another layer of chemical complexity to the system, thus giving rise to improved mechanical properties, enhanced stability, and/or additional pathways for tribofilm formation involved tribocatalytic reactions for Mo-based systems. When adding more early transition metals to the MXene structure and maintaining the overall M_4_X_3_ stoichiometry, separation/segregation effects start to occur thus enabling the formation of medium- and high-entropy MXenes, which are fully unexplored in the field of tribology.
